# A Principled Relation between Reading and Naming in Acquired and Developmental Anomia: Surface Dyslexia Following Impairment in the Phonological Output Lexicon

**DOI:** 10.3389/fpsyg.2016.00340

**Published:** 2016-03-30

**Authors:** Aviah Gvion, Naama Friedmann

**Affiliations:** ^1^Language and Brain Lab, Tel Aviv UniversityTel Aviv, Israel; ^2^Reuth Medical and Rehabilitation CenterTel Aviv, Israel; ^3^Communication Sciences and Disorders Department, Ono Academic CollegeKiryat Ono, Israel

**Keywords:** aphasia, dyslexia, surface dyslexia, Hebrew, phonological output lexicon, naming

## Abstract

Lexical retrieval and reading aloud are often viewed as two separate processes. However, they are not completely separate—they share components. This study assessed the effect of an impairment in a shared component, the phonological output lexicon, on lexical retrieval and on reading aloud. Because the phonological output lexicon is part of the lexical route for reading, individuals with an impairment in this lexicon may be forced to read aloud via the sublexical route and therefore show a reading pattern that is typical of surface dyslexia. To examine the effect of phonological output lexicon deficit on reading, we tested the reading of 16 Hebrew-speaking individuals with phonological output lexicon anomia, eight with acquired anomia following brain damage and eight with developmental anomia. We established that they had a phonological output lexicon deficit according to the types of errors and the effects on their naming in a picture naming task, and excluded other deficit loci in the lexical retrieval process according to a line of tests assessing their picture and word comprehension, word and non-word repetition, and phonological working memory. After we have established that the participants have a phonological output lexicon deficit, we tested their reading. To assess their reading and type of reading impairment, we tested their reading aloud, lexical decision, and written word comprehension. We found that all of the participants with phonological output lexicon impairment showed, in addition to anomia, also the typical surface dyslexia errors in reading aloud of irregular words, words with ambiguous conversion to phonemes, and potentiophones (words like “now” that, when read via the sublexical route, can be sounded out as another word, “know”). Importantly, the participants performed normally on pseudohomophone lexical decision and on homophone/potentiophone reading comprehension, indicating spared orthographic input lexicon and spared access to it and from it to lexical semantics. This pattern was shown both by the adults with acquired anomia and by the participants with developmental anomia. These results thus suggest a principled relation between anomia and dyslexia, and point to a distinct type of surface dyslexia. They further show the possibility of good comprehension of written words when the phonological output stages are impaired.

## Introduction

Lexical retrieval and reading aloud are often viewed as two separate processes. We draw different models for them and refer to individuals with a lexical retrieval deficit as “anomic” and to those with a deficit in reading aloud as “dyslexic”. However, these processes are not completely separate—they share components. In this study we assessed the effect of an impairment in a shared component, the phonological output lexicon, on lexical retrieval and on reading aloud.

### The lexical retrieval process and types of anomia

#### The lexical retrieval process

**Figure 1:**
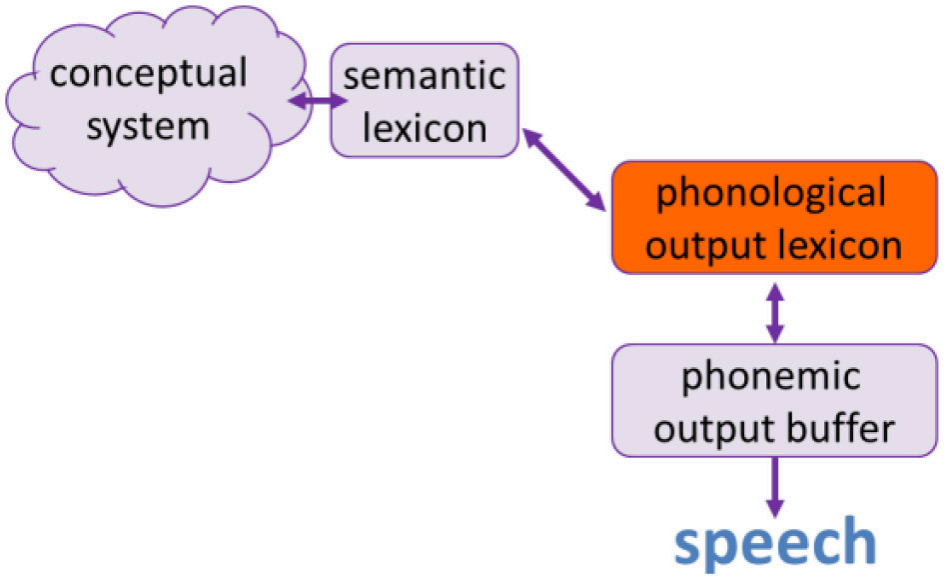
**The model for word retrieval**.

Lexical retrieval is a multi-component process, where each of the components and the connections between them can be selectively impaired and give rise to a different anomia (see Figure 1, which is a composite model based on Butterworth, 1989, 1992; Levelt, 1989, 1992; Nickels, 1997, 2002; Nickels and Howard, 2000; Friedmann et al., 2013). The first stage of the lexical retrieval process is the formation of a conceptual representation in the *conceptual system*, an a-modal representation that is still not formulated in words, which contains what the person knows about a concept, probably including its semantic properties, visual image, its function, and so on. Such concept can be created or activated from an idea someone has, or after identifying an object or event through the senses—in the case of neuropsychological assessments, usually identification of an object in a picture.

This non-lexical concept then activates a lexical-semantic representation in the *semantic lexicon* (Butterworth, 1989; Nickels, 2002; Friedmann and Biran, 2003; Biran and Friedmann, 2005, 2012)^1^. The semantic lexicon is organized semantically and contains words and information about the meaning of words. Highly imageable (concrete) words are easier to access in the semantic lexicon than low-imageability (abstract) words (Nickels and Howard, 1994; Nickels, 1995; Howard and Gatehouse, 2006). Some approaches suggest that it does not contain words as other lexicons do, but is rather a “hub” that connects the conceptual system and the various lexicons (phonological and orthographic input and output lexicons).

The selected semantic lexical entry activates the lexical-phonological representation in the *phonological output lexicon*, the protagonist of the current study^2^. The representations in the phonological output lexicon contain information about the spoken form of the word, which includes its metrical information (number of syllables and stress pattern) and its segmental information (its phonemes—consonants and vowels, and their relative positions, Butterworth, 1992; Levelt, 1992). The phonological output lexicon is organized by word frequency, and as a result high-frequency words are accessed more readily than low-frequency ones. As for the representation of morphologically complex words (at least those with regular inflections), it seems that this lexicon only includes the stems of the word, namely, it includes “orange” but not “oranges”, “smile” but not “smiled”.

The activation is in turn transferred from the phonological output lexicon to the *phonological output buffer*, a post-lexical, sub-lexical short-term memory stage. The phonological output buffer is a phonological short-term store, which holds the phonological representation that arrives from the phonological lexicon or from a sublexical route (see Section The Word Reading Process below) until the word is produced (e.g., Garrett, 1976, 1992; Kempen and Huijbers, 1983; Patterson and Shewell, 1987; Dell, 1988; Butterworth, 1989, 1992; Levelt, 1989, 1992; Nickels, 1997). This buffer holds units of various sizes: phonemes as well as pre-assembled morphemes, number words, and possibly also function words (Dotan and Friedmann, 2015). The phonological output buffer is responsible for assembling words by inserting the phonemes into the metrical frame (e.g., Meyer, 1992; Shattuck-Hufnagel, 1992; Biran and Friedmann, 2005). According to some recent studies, it is also responsible for composing morphologically complex words from their morphemes, multi-digit number names from number words, and for incorporating function words within sentences (Kohn and Melvold, 2000; Dotan and Friedmann, 2015). Given that the phonological output buffer is a short term memory component, it is affected by the length of the phonemic string it holds (namely, the number of phonemes in a word, or the number of words in a multi-digit number)—longer strings that include more units are harder to maintain and produce, and strings that include more units than the buffer can hold are impossible to maintain and produce in full.

#### Anomias: impairments in the lexical retrieval process

Anomia is a deficit in lexical retrieval, which can be acquired, i.e., occur following brain damage, or developmental—exist from birth. There exist several types of anomia, each resulting from a deficit in a different component of the lexical retrieval process (or from impaired connections between the components; Kay and Ellis, 1987; Butterworth, 1992; Nickels and Howard, 1994; Nickels, 1995, 1997, 2002; Miceli et al., 1996; Howard and Gatehouse, 2006).

*A deficit in the conceptual system* gives rise to an inability to name objects, but it has much wider repercussions: it also affects the ability to understand spoken words, written words, and even recognize and use objects, so it is quite clear that it should not be termed “anomia”. A *deficit in the semantic lexicon*, however, is a deficit that has to do with words. Because the semantic lexicon participates both in word comprehension and in word production, an anomia due to a deficit in the semantic lexicon affects both the comprehension and the production of words. Because the semantic lexicon participates in the semantics of written and spoken words, a semantic lexicon anomia affects the comprehension of both spoken and written words. Errors in naming in this type of anomia involve semantically-related word errors, as well as circumlocutions and definitions. Lexical retrieval in this anomia is affected by word imageability. Because it is a deficit in verbal processing, non-verbal material is not impaired, so pictures are understood correctly even when they are not named well; because it is a lexical deficit, the processing of non-words is unimpaired, so both reading and repetition of non-words are normal.

Phonological-lexicon anomia, an anomia that results from a *deficit in the phonological output lexicon*, affects the production of words, keeping the comprehension of words intact. Individuals with phonological-lexicon anomia make phonological- as well as semantic errors in production. When they produce semantic errors, they often comment that this is not exactly the word they were looking for. Because the phonological output lexicon is organized by frequency, these individuals show a frequency effect on naming. Given that the deficit is lexical, their non-word processing is normal.

Finally, a *deficit in the phonological output buffer* causes difficulties in word and non-word production. Errors in words and non-words are phonological; in morphologically complex words, phonological errors occur in the stems of the words, whereas the inflectional and derivational morphemes exhibit whole-morpheme substitutions and omissions. Number words in multi-digit numbers are omitted or substituted with other number words. Because the phonological output buffer is a short-term memory component, it is affected by length: stimuli with more phonemes induce more errors than shorter stimuli. Because the deficit is post the semantic stages, comprehension is intact, and no semantic errors occur. Non-words in phonological output buffer deficit are affected more gravely than words of the same length, because lexical feedback from the phonological output lexicon may support the activation of phonemes in real words but not of phonemes of non-words.

### The word reading process

**Figure 2:**
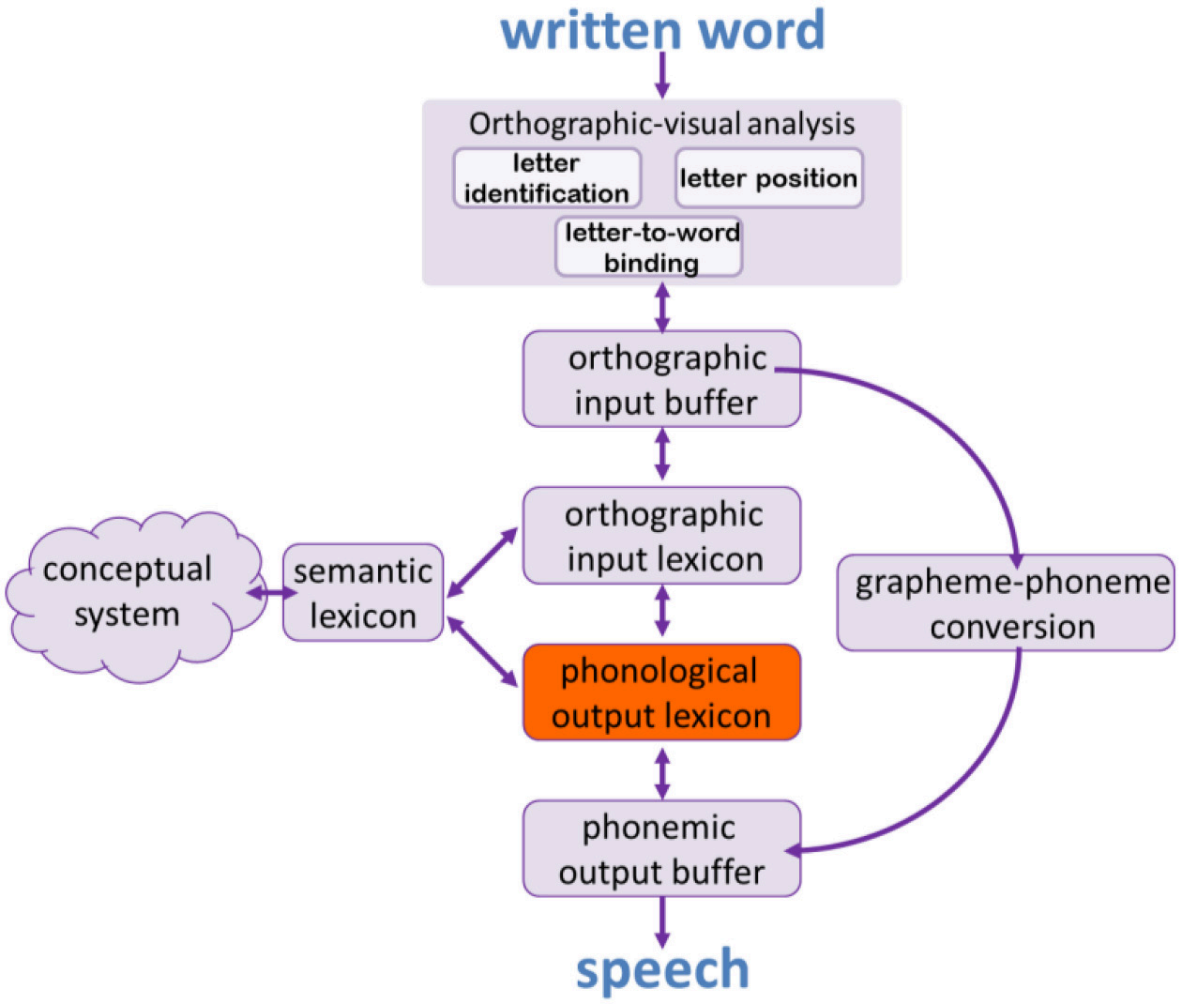
**The dual route model for single word reading**.

The word reading process, like the naming process, is also a multi-staged process, in which each of the stages and components may be affected, giving rise to a different type of dyslexia. Figure 2 presents the dual route model for single word reading (cf. Ellis and Young, 1996; Coltheart et al., 2001; Friedmann and Coltheart, 2016). The first stage of word reading, orthographic-visual analysis, is responsible for three processes: abstract letter identification, encoding of relative positions of letters within words, and binding of letters to the words they appear in (Coltheart, 1981; Ellis et al., 1987; Humphreys et al., 1990; Ellis, 1993; Peressotti and Grainger, 1995; Ellis and Young, 1996; Friedmann and Coltheart, 2016). The information from the orthographic-visual analyzer is then held in an orthographic input buffer until it flows in two routes: the lexical route, and the sublexical one.

The lexical route, which includes the orthographic input lexicon and the phonological output lexicon, allows for the accurate reading of words that the reader already knows. The orthographic input lexicon holds the orthographic information about the written form of words we know, and the phonological output lexicon, as we described above, holds the phonological information about the sounds of the spoken words we know. The direct connection between these two lexicons in the lexical route allows for a rapid and accurate conversion of a written word to its phonological form. The lexical route has another branch, which connects the orthographic input lexicon and the semantic lexicon and allows for the comprehension of written words.

The sublexical route allows for reading of new words and nonwords via the conversion of graphemes (letters or groups of letters) into phonemes. This route is typically slower and less efficient than the lexical route, and is less accurate in reading words that do not follow the grapheme-to-phoneme conversion rules.

Importantly, a look at the dual route model depicted in Figure 2 shows that the lexical retrieval process that we have described in the previous section (and in Figure 1) is actually part of the reading route: it includes all the stages between the conceptual system and the phonological output buffer (Friedmann et al., 2013). One of these shared components, the phonological output lexicon, are the topic of the current study. The phonological output lexicon is a part of the lexical retrieval process and of the lexical route for reading. As a result we expect that when the phonological output lexicon is impaired, not only lexical retrieval would be affected, but also reading via the lexical route.

### Surface dyslexia

A deficit in the lexical route is called “surface dyslexia” (Marshall and Newcombe, 1973; Coltheart et al., 1983; Newcombe and Marshall, 1985; Coltheart and Funnell, 1987; Howard and Franklin, 1987; Coltheart and Byng, 1989; Castles and Coltheart, 1993, 1996; Temple, 1997; Ellis et al., 2000; Masterson, 2000; Judica et al., 2002; Ferreres et al., 2005; Castles et al., 2006; Friedmann and Lukov, 2008, 2011). Because readers with surface dyslexia cannot read via the lexical route, they are forced to read words via the sublexical route, as if these were new words. Reading words via the sublexical route is slower than reading via the direct lexical route, and, importantly, such reading also affects reading accuracy. Some words do not obey the grapheme-to-phoneme conversion rules (e.g., words with silent letters such as *talk, walk, often*, or words in which the accurate conversion to a phoneme is the less common one, as in *door, have*). Other words include letters and letter sequences that have several options for conversion to phoneme strings (e.g., the *ea in* head, the *g* in general, Schmalz et al., 2015). These words, when read via the sublexical route, may be read incorrectly due to conversion that obeys the grapheme-to-phoneme conversion rules but is not appropriate for the target word. These errors are called “regularization errors”. A special group of irregular and ambiguous-conversion words are the *potentiophones*. These are words that, when read via grapheme-to-phoneme conversion, yield other existing words (Friedmann and Lukov, 2008). Examples for potentiophones in English are *move*, which can be sounded out via the sublexical route as “mauve”, *none*, which can be read sounding like “known”, and *phase*, which may be read like “face”. These words are especially challenging for individuals with surface dyslexia because they are not ruled out as non-words and even get feedback from the phonological output lexicon.

Regular words, in which grapheme-to-phoneme conversion rules create the correct reading, are read accurately in surface dyslexia, because they can be read correctly via the sublexical route. In addition, non-words are read correctly, because they do not need the lexical route.

#### Types of surface dyslexia

Surface dyslexia is thus defined as a deficit in the lexical route and there exist different types of surface dyslexia, depending on the component of the lexical route that is impaired (Coltheart and Funnell, 1987; Friedmann and Lukov, 2008). Deficits in each of the components of the lexical route result in reading aloud via the sublexical route and hence in inaccurate and slow reading aloud. The difference between the different types of surface dyslexia relates to the different patterns with respect to lexical decision and written word comprehension (Friedmann and Lukov, 2008). A deficit in the orthographic input lexicon affects not only reading aloud but also lexical decision (of pseudohomophones like *kloud, cranbery*, and *phun*) and comprehension of homophones (*aloud, bear, which, cite*). When the deficit in the lexical route spares the orthographic lexicon, lexical decision would be intact. When the orthographic input lexicon, the semantic lexicon, and the connection between them are intact, comprehension of homophones should also be intact.

Surface dyslexia as a result of a deficit in the orthographic input lexicon was reported in cases of acquired dyslexia (Coltheart and Funnell, 1987; Howard and Franklin, 1987; Coltheart and Byng, 1989; Weekes and Coltheart, 1996), and developmental dyslexia (Friedmann and Lukov, 2008). Additional cases of surface dyslexia that can be ascribed to the input orthographic lexicon are JC and MS, reported by Marshall and Newcombe (Marshall and Newcombe, 1973; Newcombe and Marshall, 1981, 1984, 1985). Friedmann and Lukov (2008) reported on three cases of developmental surface dyslexia as a result of an impairment to the connections of the orthographic input lexicon to the phonological output lexicon and to the semantic lexicon, and six cases with developmental impairment between the orthographic input lexicon and the phonological output lexicon.

Two interesting cases are described of people who showed surface dyslexia that can be ascribed to an impairment at the phonological output lexicon^3^. EE, the patient described by Coltheart (1982) and Howard and Franklin (1987), showed impaired naming alongside good semantic abilities (good comprehension of pictures and relatively good comprehension of auditorily presented words) and good phonological output buffer abilities (good non-word repetition and no length effect), suggesting a deficit in in the phonological output lexicon or in the connection between the semantic lexicon and the phonological output lexicon. In reading, EE showed surface dyslexia in reading aloud. The fact that he also performed poorly in input tasks involving written pseudohomophones and homophones, indicated that his surface dyslexia resulted (also) from a deficit in the input stages of reading.

EST, the patient described by Kay and Patterson in the seminal Surface Dyslexia book (Kay and Patterson, 1985) and by Kay and Ellis (1987), showed a naming pattern characterized by error types and effects on naming that are typical to impaired activation of the phonological output lexicon, alongside surface dyslexia in reading aloud: better reading of regular than irregular words, and regularization errors. His orthographic lexical judgment of pseudohomophones and his comprehension of irregular words were better than his oral reading, yet not normal. However, he also made phonological errors in non-word repetition, especially for longer non-words, and his comprehension of abstract words was impaired, suggesting that his deficit, too, was not purely at the phonological output lexicon, and may have involved the phonological output buffer and the lexical-semantic system or the access to it as well.

Kay and Ellis (1987) noticed a very interesting pattern in EST's reading: in his first reading of a word, he initially tried to read the words via the lexical route, and made phonological errors, and then moved to the sublexical route, with the result of regularization errors. It might be that his initial phonological errors resulted from a further phonological output buffer deficit.

In the current study we further explore the effect of a deficit in the phonological output lexicon on reading, with individuals with acquired or developmental anomia whose phonological output lexicon deficit was selective, and for many of whom the input reading stages and the phonological output buffer were not impaired.

### The hebrew orthography and its interaction with surface dyslexia

Hebrew, the language tested in the current study, is a Semitic language that is read from right to left. Words in Hebrew are often morphologically complex, derived from a three consonant root inserted in a derivational template and inflected for inflectional morphology. Several properties of Hebrew orthography make surface dyslexia especially noticeable and easy to detect. Vowels in the middle of the word are not consistently represented. In addition, each of the vowel letters can also be read as a consonant. Four consonant letters have ambiguous conversion to sound, and may be converted to either of two consonants. Additionally, nine phonemes can be converted to one of two or three different letters, and the stress position is lexical and not represented orthographically. These characteristics of the Hebrew orthography cause reading via the sublexical route to be error-prone, and surface dyslexia very easy to detect. In fact, there is no regular word in Hebrew, i.e., there is no word that can be unambiguously converted to a phonological string. In addition, Hebrew has many potentiophones, which makes Hebrew reading even more sensitive to surface dyslexia—for many Hebrew words, reading via the sublexical route results in another existing word so the reader cannot rule out the erroneous response based on lexicality considerations (Friedmann and Lukov, 2008, 2011). (A script that includes diacritics, *nikud*, which disambiguates most of the ambiguities in letter conversion exists but it is only used by young children in the beginning stages of reading acquisition, and in prayers and poetry).

## Participants

The participants were 16 adults and adolescents who were included in the study on the basis of their phonological output lexicon anomia. Eight of them had acquired anomia following brain damage and eight had developmental anomia. The acquired anomia group included two women and six men, and the developmental anomia group included two men and six adolescents (three girls and three boys). Background information about each of the participants is summarized in Table 1.

**Table 1:** **Background description of the participants**.

**Participant**	**Age (years)**	**Gender**	**Hand**	**Hebrew**	**Education (years)**	**Etiology**	**Lesion localization**	**Time post onset (months)**
**ACQUIRED ANOMIA**								
DAN	42	M	Right	Native	12	Anoxic brain damage	Small hypodense focus in the left caudate head	2
YOS	74	M	Right	63 years	15	Hydrocephalus		2
ZAB	77	M	Right	64 years	12	Stroke	Basal ganglia infarct	4
BAR	57	F	Right	39 years	15	Stroke	–	2
LER	78	M	Right	native	12	4th stroke	Left parieto-occipital stroke	3
ARI	67	M	Right	Native	12	Stroke	Left fronto-temporal-occipital infarct	48
NAV	72	F	Right	Native	15	Stroke	Infarct in the thalamus and left internal capsule	1
DOR	55	M	Right	Native	17	Encephalitis	Left temporal lesion	17
**DEVELOPMENTAL ANOMIA**								
**Participant**	**Age (years; months)**	**Gender**	**Hand**	**Hebrew**	**Education (current grade/years)**			
LEO	28;8	M		24 years	16 (MA student; a teacher)			
TAF	49;3	M		20 years	17 (MA)			
AFI	16;6	F	left	Native	9th grader			
ARO	10;4	M	left	Native	4th grader			
MAD	12;6	M		Native	7th grader			
SAN	13;1	F	left	Native	7th grader			
NIV	12;8	M	left	Native	7th grader			
SHL	12;9	F	right	Native	7th grader			

Four of the participants with developmental anomia were of the same family: TAF was the father of AFI, MAD, and ARO (an additional daughter had only a mild anomia and was therefore not included in the study). All participants, including the four who immigrated to Israel after the beginning of elementary school, reported that Hebrew was the main language they used for reading and writing.

The participants were selected from a pool of individuals with acquired or developmental language deficits who were complaining of naming difficulties and were referred to a rehabilitation center in central Israel or to our Language and Brain Lab at Tel Aviv University.

## General procedure

Each participant was tested individually in a quiet room. During the testing sessions, the experimenter wrote down every response that differed from the target. All the sessions were audio-recorded and two judges listened to the recordings after the sessions, and checked the transcriptions from the session against the recordings, completing and correcting them when necessary. The pictures and the written stimuli of the various tests were presented to each participant over the desk, printed on a white page. In the oral reading tasks, the participant was requested to read aloud as accurately as possible; in the lexical decision and comprehension tasks (see Section Reading Tests), the participant was requested to perform the task without reading aloud. According to the availability for testing of each of the participants, some of them were tested with more tests from the battery, and some with fewer tests—the results of each test for each participant appear in the tables below. No time limit was imposed during testing, and no response-contingent feedback was given by the experimenter, only general encouragement. The participants were told that whenever they needed a break they could stop the session or take a break.

### Data analysis

To compare the performance of each experimental participant to her/his age-matched control group, we used Crawford and Howell's (1998) *t*-test, and reported that an individual performed significantly below the control group when *p* < 0.05 in this test^4^. The children were compared to control groups not only by age but also by grade level, and all the adult participants in all the control groups had 12 years education and above, as did the anomic participants. The effects on word retrieval (length, frequency) were calculated as the point biserial correlation between the word property and the success in producing the target word. An alpha level of 0.05 was used in all analyses.

## Assessment of lexical retrieval: testing to establish phonological output lexicon deficit for inclusion in the study

### Tests establishing a phonological output lexicon deficit

In order to establish that a participant had a phonological output lexicon deficit and could be included in our study, we started by testing picture naming for all participants. Those who showed impaired naming that could result from a phonological output lexicon deficit received additional tests to assess the exact locus of their impairment in the lexical retrieval process. These tests included conceptual tests; repetition of words, pseudo-words, and morphologically complex words; comprehension of heard and written words; reading aloud of Arabic numbers; and phonological short-term memory tests.

#### Naming task

***Picture naming*** was assessed using the *SHEMESH* test (Biran and Friedmann, 2005), which includes 100 color pictures of objects of various semantic categories. The target nouns were feminine and masculine nouns, morphologically simple and complex, with regular and irregular gender morphology, 1–4 syllable long, 3–10 phonemes, with ultimate and penultimate stress and with various first phonemes.

The frequency of the target words, judged by 75 Hebrew-speaking participants with no language deficits, ranged from 2.39 to 6.84 on a scale of 1–7 (*M* = 4.90, *SD* = 1.09). The performance of Hebrew speakers without a language deficit in this test is very high (average 95.6% correct, *SD* = 4.2%, for 67 control subjects aged 50–80; average 98.7% correct, *SD* = 1.7%, for 87 control subjects aged 20–40; and 94.1% correct, *SD* = 2.3%, for 35 control subjects aged 12–14, Biran and Friedmann, 2004, 2005).

#### Additional tasks to establish a phonological output lexicon deficit and exclude impairments at other levels

A naming deficit that results from a deficit in the phonological output lexicon should not affect semantic and conceptual abilities, nor should it impair non-word processing. We thus tested these abilities, using several additional tasks.

*The conceptual system* was tested using a ***picture association test*** (MA KASHUR pictures, Biran and Friedmann, 2007). This task includes 35 triads of pictures, a target object presented at the top (e.g., *cow*) and two pictures at the bottom, one semantically related to the target picture (e.g., *milk*) and one unrelated but from the same category or associated with the other picture on the bottom (e.g., *Coca-Cola*). The participants are requested to choose the picture that is semantically related to the top picture.

*The semantic lexicon* and the access to it from written words were tested using the verbal counterpart of the picture association task, the ***written word association test*** (MA KASHUR words, Biran and Friedmann, 2007)^5^. This task includes 35 triads of written words. Of these, 25 are identical to 25 of the pictorial triads, and 10 triads include abstract terms (e.g., honesty–truth/lie).

An additional task that we used to examine the semantic lexicon was *a* **spoken word-to-picture matching task** from the Psycholinguistic Assessment of Language Processing in Aphasia (PALPA 47, Kay et al., 1992; Hebrew version Gil and Edelstein, 2001). This test consists of 40 groups of five pictures including a target word (e.g., *a dog*) and four close and distant semantic distracters (e.g., a cat, a giraffe, a rocking horse, and a kite, respectively). The participants are requested to select the picture that matches the word they heard.

The *phonological output buffer* was assessed using ***a non-word repetition test*** (*BLIP*, Friedmann, 2003). The participants were requested to repeat 48 non-words that the experimenter said. The test includes 24 easy non-words of 2, 3, and 4 CV syllables (8 of each length), and 24 phonologically complex non-words (of 2, 3, and 4 syllables) with clusters in various word position or with phonological feature similarity.

A phonological output buffer impairment also affects the production of morphologically complex words and multi-digit numbers (Dotan and Friedmann, 2015). Therefore, as another tool to assess a phonological output buffer impairment we administered a test of ***repetition of morphologically complex words*** (the MURKAMOT test, from the Buffy battery, Friedmann, 2006). This test consists of 36 morphologically complex words, 24 of the words included a stem/root and inflectional or derivational morphemes (half with 1 morpheme and half with 2), and 12 were long morphologically-simple words. (LER and ZAB were tested using a short version of the non-word and morphological complex repetition tests that included only 10 items each).

Multi-digit number processing was tested using a task of ***oral reading of multi-digit Arabic numbers***, which included 60 numbers pf 2–5 digits, 15 numbers of each length.

Additional tests for the phonological output buffer included phonological STM tasks from the *FriGvi* battery (Friedmann and Gvion, 2002; Gvion and Friedmann, 2012). These included *a* ***basic word recall span*** test that tests the recall of sequences of 2–7 phonologically different two-syllable words (five word sequences in each length); a ***long word recall span*** test, with sequences of 2–7, phonologically different four-syllable words (five word sequences in each length); and a ***non-word recall span*** testing sequences of 2–7, two-syllable non-words, constructed by changing a single consonant in real words (five non-word sequences in each length). To measure the participants' input span in a task that does not involve speech output, and allow for the comparison between span tasks with and without overt speech in order to evaluate the input and output buffers separately, we administered to some of the participants a recognition STM task, the ***matching word order span***. In this task, the participants heard, in each item, two sequences of 2–7 words containing the same words (2-syllable phonologically dissimilar words, 10 items per length) either in the same or in a different order, and were asked to judge whether the order of the items in the two lists was the same. On the non-identical pairs, the two lists differed in the order of two adjacent words. The span level is defined as the maximal level at which the participant performed correctly on at least 7 of 10 items.

### Results: lexical retrieval performance and locus of deficit

#### Acquired anomia

The performance of the individuals with acquired anomia on the picture-naming test is summarized in Table 2. As demonstrated in the table, the performance of each of them was significantly below that of their age-matched control groups at a level of *p* < 0.0001. The participants named correctly 21%–81% of the pictures, with an average of 53.8% (SD = 22%) correct.

**Table 2:** **Picture naming: %correct, error types, and effects–acquired anomia**.

	**DAN**	**YOS**	**ZAB**	**BAR**	**LER**	**ARI**	**NAV**	**DOR**
**% Correct naming**	68%^***^	81%^***^	56%^***^	73%^***^	41%^***^	21%^***^	62%^***^	28%^***^
**Error types**								
Phonologically related non-word	27%		2%	3%	43%	10%		
Phonologically related word	2%				18%	10%	5%	
Phonological approximations	18%		7%		3%	45%	15%	34%
Hesitations, long latency	27%	35%	57%	35%	11%	1%	42%	28%
Paraphrases, definitions	9%	61%	12%	24%	3%	8%	25%	23%
Semantically related word	13%		5%		5%	1%		1%
Morphological error					1%		2%	2%
No response/don't know	2%		7%			7%		1%
Naming in another language				29%				
Related gesture			10%	6%	4%			9%
Superordinate category							2%	2%
Unrelated syllables						14%		
Neologism	2%				1%	1%	2%	
Perseveration		4%		3%	11%	3%	7%	
Frequency effect	*r* = 0.27, *p* = 0.003	*r* = 0.15, *p* = 0.07	*r* = 0.20, *p* = 0.02	*r* = 0.28, *p* = 0.002	*r* = 0.15, *p* = 0.08	*r* = 0.23, *p* = 0.01	*r* = 0.15, *p* = 0.07	*r* = 0.20, *p* = 0.02
Length effect	*r* = −0.17, *p* = 0.05	NO	NO	NO	*r* = −0.19 *p* = 0.03	*r* = −0.15, *p* = 0.07	NO	NO

*The percentages in the error type cells represent percentage of the total number of errors the participant made*.

*** *Comparison of percentage correct of naming of each participant to his/her matched control group, p < 0.001*.

To examine the locus of the deficit in the lexical retrieval process of each of the participants, and to establish whether they have a phonological output lexicon impairment, we used three criteria: Error types, effects on naming, and performance on the other, semantic and phonological tasks.

##### Error types

As shown on Table 2, the error pattern of each of the participants was the one typical of phonological output lexical impairment: hesitations and long response latencies (*M* = 29.4%, *SD* = 17%), relevant paraphrases (*M* = 20.6%, *SD* = 18%), phonological approximations (*M* = 15.3%, *SD* = 17%), phonologically-related words and non-words (*M* = 7.5%, *SD* = 12%), and semantically-related words, usually followed by self-correction attempts (*M* = 3.1%, *SD* = 5%). Other types of errors were relatively few.

##### Effects on naming

As shown on Table 2, the typical **frequency effect** on naming, with higher frequency words named better than lower frequency ones, was significant for five of the participants (DAN, ZAB, BAR, ARI, DOR), and marginally significant for the other three (YOS, LER, NAV). LER also showed a significant length effect, and DAN and ARI showed a marginally significant one.

##### Performance in other tasks

The performance of each of the individuals with acquired anomia in the additional semantic and phonological tests is summarized on Table 3. The tests that assessed their semantic-conceptual abilities, which included picture-picture, word-picture, and word-word matching tasks, indicated that the lexical-semantic and conceptual levels of each of the participants are preserved. Seven of the eight participants were tested using the word-word association test, which assessed the lexical-semantic level (as well as the conceptual system), where they all performed at least 95% correct. One patient was tested only using the picture association task, on which he also performed 95% correct.

**Table 3:** **%Correct performance of the individuals with acquired anomia in tasks that involve conceptual, lexical semantics, and phonemic output buffer**.

**Tasks**	**DAN**	**YOS**	**ZAB**	**BAR**	**LER**	**ARI**	**NAV**	**DOR**
**CONCEPTUAL AND SEMANTIC TASKS**								
Picture association	95%		100%			97%		
Written word association^a^		100%	100%	95%^*^	100%	100%	100%	98%
Word to picture matching				95%	88%			
**PHONOLOGICAL BUFFER TASKS**								
Non-word repetition				100%	30%^*^	29%^*^	100%	100%
Morph word repetition			90%		90%		100%	
Word repetition	66%^*^						100%	
Arabic number reading			97%		81%^*^	85%^*^		
Basic word span	2^*^	4				2^*^		4
Non-word span						1.5^*^		2^*^
Matching word order span	4^*^		5			7		

a *The scores for ZAB and ARI refer to their performance in the written word association test (Biran and Friedmann, 2007). For the other participants the scores refer to the homophone-potentiophone written comprehension test*.

* *Significantly below the control group, p < 0.05*.

In the tests that assessed their phonological (input and) output buffer, five of participants with acquired anomia (YOS, ZAB, BAR, NAV, and DOR) showed good performance, and three (DAN, LER, and ARI) showed indications of an additional impairment in the input and/or output phonological buffers. On the basis of their performance in the span tasks, ARI probably had a deficit in the phonological output buffer, as his recognition span was within matched controls range, whereas DAN's limited input span suggests that he also has a phonological input buffer deficit, which may have contributed to his difficulty in word repetition. His phonological output buffer was also impaired, as indicated by the length effect he demonstrated in naming (Table 2). (DOR's non-word span was 0.5 words below the normal range, but given his 100% correct repetition in the difficult non-word repetition task, and his normal word span, we considered his phonological buffers unimpaired).

Thus, the error pattern, which is typical of a deficit at the phonological output lexicon, as well as the frequency effect and the performance on semantic and phonological tasks, indicate that the anomia of all eight participants with acquired anomia resulted from a deficit in the phonological output lexicon. Three of them (DAN, LER, and ARI) probably had an additional deficit in the phonological output buffer. (We conclude that they had a phonological output buffer deficit in addition to a phonological output lexicon and not only a phonological output buffer deficit on the basis of the frequency effect they showed in naming, as well as on the basis of the semantic errors that they made in picture naming, which cannot be explained by a phonological output buffer, and cannot be ascribed to impaired semantic-conceptual system, because they all demonstrated good lexical-semantic and conceptual abilities).

#### Developmental anomia

The results of the naming test of the individuals with developmental impairments, including the rate of correct responses, types of errors, and effects on naming, are summarized in Table 4. Each of the participants performed significantly below her/his age-matched group in the naming test, *p* < 0.001. They named between 68% and 85% of the pictures correctly.

**Table 4:** **Picture naming test: Correct performance, error types, and effects–developmental anomia**.

	**LEO**	**TAF**	**AFI**	**ARO**	**MAD**	**SAN**	**NIV**	**SHL**
**% Correct**	80%^***^	76%^***^	85%^***^	71%^***^	68%^***^	85%^***^	80%^***^	84%^***^
**Error types**								
Phonologically related non-words		5%			3%	18%	6%	
Phonologically related words					3%	12%		8%
Phonological approximations					5%	12%		
Hesitations, long latency	44%	45%	33%	23%	57%	40%	17%	54%
Paraphrases, definitions	13%		6%	8%		6%		8%
Semantically related word	4%		17%		5%	12%	50%	15%
Morphological error		14%			3%			
No response/don't know		36%		50%			27%	15%
Naming in another language	4%		38%	19%	24%			
Related gesture	31%		6%					
Superordinate category	4%							
Frequency effect	NO	*r* = 0.34, *p* = 0.0003	*r* = 0.4, *p* = 0.001	*r* = 0.31, *p* < 0.0001	*r* = 0.3, *p* = 0.003	*r* = 0.2, *p* = 0.04	*r* = 0.25, *p* = 0.006	NO
Length effect	NO	NO	NO	NO	NO	NO	*r* = −0.17, *p* = 0.04	NO

*The percentages in the error type cells represent percentage of the total number of errors the participant made*.

*** *Comparison of percentage correct of naming of each participant to his/her matched control group, p < 0.001*.

##### Error types

Similarly to the participants with acquired anomia, the t*ypes of errors* that the participants with developmental anomia made were the typical errors evinced in phonological output lexicon anomia: hesitations and long response latencies (*M* = 39.4%, *SD* = 14.3%), no responses or “don't know” responses (*M* = 16.2%, *SD* = 19.8%), semantically related words, usually followed by self-correction attempts (*M* = 12.7%, *SD* = 16.5%), naming in another language (*M* = 11.4%, *SD* = 15.6%), relevant definitions and circumlocutions (*M* = 5%, *SD* = 4.7%), related gestures (*M* = 4.5%, *SD* = 10.7%), and phonologically-related words and non-words (*M* = 3%, *SD* = 5%). Other types of errors were relatively few.

##### Effects on naming

Six of the participants (TAF, AFI, ARO, MAD, SAN, and NIV) manifested the typical *frequency effect* (*p* ≤ 0.04). NIV also showed a length effect (*p* = 0.04). Two other participants (LEO, SHL) were not affected by either frequency or length effects.

##### Performance in other tasks

The performance of each of the individuals with developmental anomia in the semantic and phonological tests is summarized on Table 5. The good performance of the developmental anomic participants on the conceptual and lexical-semantic tests indicates that their semantic lexicon and conceptual system are preserved.

**Table 5:** **%Correct performance in tasks of conceptual, lexical semantics and phonemic output buffer–developmental anomia**.

**Task**	**LEO**	**TAF**	**AFI**	**ARO**	**MAD**	**SAN**	**NIV**	**SHL**
**CONCEPTUAL AND SEMANTIC TASKS**								
Picture association			100%	100%	100%			
Written word association^a^	98%	100%	100%	100%	100%	93%	95%	100%
Word-picture matching				100%	100%			
**PHONOLOGICAL BUFFER TASKS**								
Non-word repetition	100%	92%	98%	96%	91%	58%^*^	90%	73%^*^
Morph word repetition				100%			94%	100%
Basic word span	6.5		5	4.5		3^*^	3^*^	2.5^*^
Long word span			4	3.5	4			
Non-word span			3			0^*^		2^*^
Matching order word span			7			4		3^*^

* *Significantly worse than age-matched control group (p < 0.05)*.

a *The scores for ARO and MAD refer to their performance in the written word association test (Biran and Friedmann, 2007). For the other participants the scores refer to the homophone-potentiophone written comprehension test*.

All but two of the developmental anomic participants performed well on the non-word repetition task, indicating well-functioning input and output phonological buffers. Two girls (SAN and SHL), however, performed poorly on repetition of non-words. SHL showed impaired performance on the input span task, and did not show a length effect in naming, so her poor non-word repetition may be attributed to a limited phonological input buffer, in addition to phonological output lexicon deficit. SAN's poor non-word repetition is a bit more difficult to interpret, as her input span was within the normal range for her age, indicating intact phonological input buffer, but she also did not show length effect in naming, which casts doubt on a deficit in the phonological output buffer.

The naming of one participant (NIV) was affected not only by frequency effect but also by length effect. Length effect could indicate a phonological output buffer impairment, but given that his repetition of non-words and morphologically complex words was relatively spared, it seems that he does not have a phonological output buffer deficit on top of his phonological output lexicon impairment.

Thus, based on typical errors, effects on naming, and the performance in semantic and phonological tasks, like the participants with acquired anomia, all eight participants with developmental anomia have a deficit in the phonological output lexicon. Although two of the developmental anomic participants did not manifest the expected frequency effect in naming, their performance in other tasks and the types of errors they made in naming imply that their deficit is at the phonological output lexicon. Two of the participants may have also had a phonological input or output buffer deficit, in addition to their phonological output lexicon deficit.

## How does a deficit at the phonological output lexicon affect reading?

In order to test our main research question for this study, the way a deficit in the phonological output lexicon affects reading, we assessed the participants' oral reading, as well as their performance in reading tasks that do not involve speech output and hence, do not involve the phonological output lexicon. For assessing oral reading, we administered a word reading aloud test that includes single words of various types, including irregular and potentiophonic words, and an additional test of oral reading of potentiophones, which are particularly sensitive to surface dyslexia. To evaluate the earlier, input stages of reading through the lexical route—the orthographic input lexicon and its connection to the semantic lexicon, we used an orthographic lexical decision task and a task that assessed the comprehension of written homophones and potentiophones.

### Reading tests

#### Oral reading tasks

##### The TILTAN oral reading screening test (Friedmann and Gvion, 2003)

The screening test served two purposes: to examine whether the participants had surface dyslexia, by assessing their oral reading of irregular and potentiophonic words, and to test whether they had any other types of dyslexia, apart from surface dyslexia.

The screening test includes 136 single Hebrew words that were constructed so that they are sensitive to the various types of dyslexia: 65 migratable words, to detect letter position dyslexia; All the words in the test are sensitive to left neglect dyslexia at the word level, as all the words in the list are such that neglect errors on their left side yield other words; 104 of the words are sensitive to right neglect, as neglect errors on their right side create other existing words; 89 abstract words, for identifying deep dyslexia; function words and morphologically complex words, for identifying deep dyslexia and phonological output buffer dyslexia; words with many orthographic neighbors for identifying visual dyslexia; and words for which migrations, substitutions, omissions, or additions of a vowel letter create other existing words for identifying vowel letter dyslexia (Khentov-Kraus and Friedmann, 2011).

Most importantly for our study, the test includes words for identifying surface dyslexia. In Hebrew, as we explained above (Section The Hebrew Orthography and Its Interaction with Surface Dyslexia), there are no words that can be read unambiguously and correctly through grapheme-to-phoneme conversion. Therefore, essentially all words in the screening test are sensitive to surface dyslexia^6^. The test also included 35 potentiophones, which are most sensitive to surface dyslexia, and 33 irregular words that are parallel to irregular words in English—words with silent letters or with ambiguous letters that are converted to the less common rendition of the letter.

##### Potentiophone reading test

Potentiophone reading test (also from the TILTAN battery, Friedmann and Gvion, 2003).

To assess directly the participants' ability to read via the lexical route, we tested their reading of the stimuli that are most sensitive to sublexical reading: potentiophones. The potentiophone test includes 78 potentiophonic words, 2–6 letters long (*M* = 3.7 letters, *SD* = 0.8).

#### Reading tasks without oral output

We used two tasks to examine the way the participants process pseudohomophones, homophones, and potentiophones when they were requested to avoid oral production and hence. This allowed us to examine how they read when their impaired phonological output lexicon is not involved.

##### Written lexical decision (Friedmann and Lukov, 2008)

To assess the orthographic input lexicon and the access to it, we tested the participants' ability to decide whether a pseudohomophone is a word or not, using a visual-word recognition task of lexical decision, which proved sensitive to surface dyslexia with orthographic input lexicon deficit (Friedmann and Lukov, 2008). The test consisted of 68 pairs, each pair includes a correctly spelled word (*shoe*) and its pseudohomophone (*shoo*). Twelve of the words were irregular (including a silent letter or a letter that is the less frequent orthographic representation of the phoneme), and the parallel pseudohomophone was the regular spelling of the word (e.g., *school-scool*). The other pairs included words in which at least one phoneme can be ambiguously converted to a letter (e.g., *city-sity*). The participants were requested to circle the correctly spelled word. The control groups for this test included 148 adult participants aged 20–72, with 12 years education and above—like the anomic participants, and 201 children and adolescents in 4th–9th grade (see **Table 7**).

##### Written homophone-potentiophone comprehension (Friedmann and Lukov, 2008)

To examine the participants' access from the orthographic input lexicon to the semantic lexicon (as well as the status of the orthographic and semantic lexicons themselves), we tested the comprehension of homophones or potentiophones. The test consisted of 40 triads, each triad includes a target word (e.g., pay), and two additional words. One word was semantically related to the target word (buy), the other word was a homophone or a potentiophone of the related word (bye). Twenty of the target words were abstract (the target word was of low imageability), and 20 target words were concrete nouns or verbs. Each participant was requested to find the word that is semantically associated with the target word, and draw a line between them. This test, too, was used in previous surface dyslexia studies and proved sensitive to surface dyslexia with input deficit (Friedmann and Lukov, 2008). The control groups for this test included 141 adult participants aged 20–70, and 169 children and adolescents in 4th–9th grade (see **Table 7**).

### Results: oral reading tasks

Table 6 summarizes the performance of each of the participants in the oral reading tests. The results indicate that all the 16 anomic participants with a phonological output lexicon deficit had surface dyslexia in reading aloud—namely, their oral reading indicated that they were using the sublexical, rather than the lexical route for reading aloud.

**Table 6:** **Oral reading tasks: %correct and number of surface errors**.

**Participant**	**Single words TILTAN screening**		**Potentiophones**	
	**%Correct**	**Surface errors**	**%Correct**	**Surface errors**
**ACQUIRED ANOMIA**				
DAN	63%^***^	16^***^	58%^***^	32^***^
YOS	92%^***^	8^***^	81%^***^	12^***^
ZAB	85%^***^	6^***^	72%^***^	17^***^
BAR	93%^***^	4^**^	82%^***^	14^***^
LER	55%^***^	12^***^	51%^***^	25^***^
ARI	74%^***^	25^***^	60%^***^	31^***^
NAV	94%^***^	8^***^	87%^***^	10^**^
DOR	88%^***^	12^***^	83%^***^	15^***^
**DEVELOPMENTAL ANOMIA**				
LEO	93%^***^	7^***^	78%^***^	17^***^
TAF	74%^***^	27^***^	51%^***^	37^***^
AFI	96%	2	79%^*^	16^*^
ARO	38%^***^	64^***^		
MAD	85%^***^	17^***^	53%^***^	36^***^
SAN	81%^***^	9^***^		
NIV	82%^***^	12^***^		
SHL	81%^***^	12^***^		
**Control groups M (SD)**				
Adults, 12 years education and above *N* = 372	98.4% (1.4)	1.1 (1.2)	94.7% (3.6)	3.7 (2.7)
7th grade control *N* = 26	96.0% (1.4)	3.2 (1.7)	90.4% (4.6)	7.1 (3.7)
5th grade control *N* = 14	96.4% (2.5)	2.7 (1.9)	88.8% (6.7)	8.4 (5.0)
4th grade control *N* = 20	90.6% (5.4)	7.2 (5.1)		

*Significantly more errors than age-matched control group*,

* *p ≤ 0.02*,

** *p ≤ 0.01*,

*** *p ≤ 0.001*.

All the participants, those with acquired anomia and those with developmental anomia, performed significantly below the age-matched control readers in reading aloud of the single words in the screening test and of the potentiophone word list, and each of them made significantly more surface errors than their age-matched peers (one participant, AFI, had significantly more surface errors than the control group only in the potentiophone list). Their surface errors were the errors we typically see in the reading aloud of Hebrew-readers with surface dyslexia: reading the target word in a way that is a plausible reading according to grapheme-to-phoneme conversion rules, including errors of stress position, as the stress in Hebrew is not marked lexically, errors of the choice of vowels that are not marked orthographically, reading silent letters, and converting of a grapheme that has several possible conversions to a phoneme that is a possible conversion but not the right one for the target word.

Importantly, most of their errors in reading aloud were surface errors, namely, errors that were phonologically acceptable conversions of the target words, but which indicated that the words were not read via the lexical route. The number of surface errors in the screening task and in the potentiophone task is presented in Table 6,^7^.

The oral reading of YOS, ZAB, NAV, DOR, LEO, TAF, AFI, and MAD was selectively impaired, and the pattern was that of a pure surface dyslexia. Some other participants (mainly DAN, LER, ARI, ARO, SAN, NIV, and SHL) showed a clear surface dyslexia but also made additional types of errors in oral reading, including letter migrations, substitutions, omissions, and additions, which resulted from their letter position dyslexia or attentional dyslexia (ARO, SAN, NIV, and SHL) or from a phonological output buffer deficit (DAN, LER, and ARI). See Appendix A in Supplementary Material for a detailed presentation of all errors types each of the participants made in each of the reading aloud tests, and for further assessment of the additional dyslexias of these seven patients.

### Results: input reading tasks

We have seen that, when asked to read aloud, all the participants with phonological lexicon impairment read via the sublexical route, and therefore show a pattern that is characteristic of surface dyslexia. Does it indeed result, as we have suggested, from their phonological output lexicon deficit, or do they have a deficit in the orthographic input lexicon, which causes their sublexical reading? We examined this question by assessing their reading in tests that did not involve oral production.

The results of these tests, summarized in Table 7, indicated that most of the participants performed very well and not differently from age- (and grade-) matched controls when the reading task did not involve output. All but the two youngest children performed at a level of 93% correct and above in both tasks^8^.

**Table 7:** **Lexical decision and comprehension tasks: %correct**.

**Participant**	**Lexical decision**	**Homophone-potentiophone comprehension**
**ACQUIRED ANOMIA**		
DAN	96%	95%
YOS	100%	100%
ZAB	93%^**^	93%^*^
BAR	100%	95%
LER	100%	100%
ARI	99%	93%^*^
NAV	100%	100%
DOR	98%	98%
**DEVELOPMENTAL ANOMIA**		
LEO	100%	98%
TAF	100%	100%
AFI	100%	100%
ARO	75.7%^*^	74%
MAD	89%^**^	90%^*^
SAN	96%	93%
NIV	100%	95%
SHL	96%	100%
**CONTROL GROUPS M (SD)**		
Adults, at least 12 years education	98.0% (1.5) *N* = 148	98.3% (2.4) *N* = 141
6th–9th grade control groups	9th grade	7–9th grade
	99.3% (1.1) *N* = 59	97.4% (2.7) *N* = 74
	7–8th grade	
	98.9% (2.7) *N* = 24	
	6th grade	
	97.4% (2.3) *N* = 10	
5th grade control group	98.6% (1.8) *N* = 28	94.2% (4.7) *N* = 18
4th grade control group	93.8% (10.1) *N* = 80	87.9% (11.6) *N* = 77

*Significantly more errors than age-matched control group*,

* *p < 0.05*,

** *p = 0.001*.

Thus, the performance of the participants with impaired phonological output lexicon on the reading tasks indicates that they show a reading pattern of surface dyslexia in oral reading, but not in orthographic lexical decision and written comprehension tasks that do not involve oral reading. This indicates that these individuals rely on the sublexical route when they need to read words aloud, and that this does not result from a deficit in the orthographic input lexicon.

This pattern, of sublexical reading aloud with preserved orthographic input lexicon and access from it to the semantic system, applied both to the participants with acquired phonological output lexicon impairment and to those with a developmental phonological output lexicon impairment.

Recall that we selected the participants to this study solely on the basis of their naming deficit, which results from a phonological output lexicon impairment. We only then tested their reading patterns. Given that all these participants showed surface dyslexia in reading aloud, we can conclude that the phonological output lexicon deficit causes surface dyslexia in reading aloud, and that it can occur alongside good performance in tasks that do not involve speech output. Some of the participants, and particularly the youngest developmental anomic MAD and ARO, may have also had an orthographic input lexicon impairment or at least have not yet established a rich enough set of lexical entries in this lexicon, on top of their phonological output lexicon impairment. Importantly, however, the fact that there were 12 participants who showed completely normal performance in the input reading tasks indicates that phonological output lexicon impairment can cause a very selective surface dyslexia, which only affects reading aloud.

## Discussion

### Phonological output lexicon deficit causes surface dyslexia

Lexical retrieval and reading are often depicted using different models, and studied by different researchers. However, the current study demonstrated that they are tightly linked. We focused on a component that is part of both lexical retrieval and reading aloud: the phonological output lexicon. Our main finding is that individuals with acquired or developmental anomia that results from a deficit in the phonological output lexicon also show a very clear and consistent deficit in reading: when they read aloud they make regularization errors in irregular words, indicating reading via the sublexical route, but when their silent reading is tested, in tests of lexical decision and written words comprehension, which do not involve phonological output, they perform normally. A look at the models of reading and lexical retrieval explains exactly why this is so: the phonological output lexicon is part of the lexical route for reading aloud, and its impairment results in reading aloud via the sublexical route. However, because the deficit is only located in a late, output stage of reading, their input, including the orthographic input lexicon, is not impaired, and this is what allows them to judge correctly pseudo-homophones as non-words, and to understand written words well, including homophones and potentiophones. This pattern held for individuals with various sources of phonological lexicon anomia: acquired and developmental, for individuals in different ages and levels of education. This indicates that this strong relation between phonological output lexicon anomia and surface dyslexia occurs independently of specific source of impairment. The fact that the individuals with developmental anomia showed the same pattern as the individuals with acquired anomia also suggests an interesting insight about reading acquisition. It suggests that entrees in the orthographic input lexicon and their connection to the semantic lexicon can be established even when the phonological output lexicon is impaired. Namely, the orthographic input lexicon can be established even without well-functioning reading aloud.

### The road not taken

Given that the phonological output lexicon is part of the lexical route, two possibilities are imaginable for the way a deficit in the phonological output lexicon may affect reading: one is that reading via the lexical route is blocked and hence reading has to proceed via the sublexical route, giving rise to surface dyslexia. The other is that the reader with phonological output lexicon would still use the impaired lexical route for reading aloud, and this would result in phonological errors in reading aloud that are similar to the errors made in speech production. Our results from the participants who had a selective deficit in the phonological output lexicon indicate that they only read via the sublexical route, and the other theoretically possible option is not attested: they do not read via the impaired lexical route, and do not make phonological errors in reading aloud. There were five participants in the current study who did make errors in reading beyond surface dyslexia errors that could be phonological errors. Importantly, such errors occurred only in the five participants who had, in addition to their phonological output lexicon impairment, also an impairment in the phonological output buffer. Their phonological errors in reading, thus, can be ascribed to the later, phonological output buffer deficit and not to reading via the lexical route. This may also explain the pattern of errors reported for Friedman and Kohn's (1990) patient HR: HR had impaired phonological output in naming, and in reading aloud he made phonological errors. On the basis of his impaired processing of non-words and the length effect he showed in all production tasks, one may conclude that his deficit did not lie at the phonological output lexicon but rather in the phonological output buffer, and this was the source of his phonological errors in reading aloud.

Our results suggest another type of surface dyslexia, which occurs both in acquired dyslexia and in developmental dyslexia: surface dyslexia that results from a deficit in the phonological output lexicon (see also EST and EE in Coltheart, 1982; Kay and Patterson, 1985; Howard and Franklin, 1987; Kay and Ellis, 1987, for earlier cases of acquired phonological anomia and surface dyslexia, albeit with a less selective pattern). This type of surface dyslexia joins other types of surface dyslexia that have been reported: a selective deficit in the orthographic input lexicon, a deficit in the output of the orthographic input lexicon (to the phonological output lexicon and to the semantic lexicon), and an inter-lexical deficit between the orthographic input lexicon and the phonological output lexicon (Coltheart and Funnell, 1987; Friedmann and Lukov, 2008).

Given the consistent effect the deficit in the phonological output lexicon had on the reading of the participants, regularization errors in reading aloud may be taken in the future as another tool for the functional localization of the source of anomia in the lexical retrieval process. It is often difficult, for example, to distinguish between a deficit in the connection between the semantic lexicon and the phonological output lexicon and a deficit in the phonological output lexicon itself. Our findings suggest a way to distinguish between the two, as a deficit in the connection between the semantic lexicon and the phonological output lexicon should not cause surface dyslexia (given that a direct connection between the orthographic input lexicon and the phonological output lexicon is still available for reading aloud)^9^, but phonological output lexicon deficit should.

The identification of this shared destiny between lexical retrieval and reading impairments also has clinical implications: when a person has a deficit in the phonological output lexicon, either due to brain damage or from birth, one may expect this person to have difficulties in oral reading as well. Treatment of the lexical retrieval difficulty is thus expected to also reduce errors in reading aloud. Importantly, these difficulties in reading only affect reading aloud. These people can still understand and recognize written words very well. Therefore, the clinician can provide a very straightforward recommendation to these individuals: do not read aloud.

## Author contributions

The authors conceived the research question together, collected the data together, analyzed the data together, thought together about the results and their implications, and wrote the paper together.

### Conflict of interest statement

The authors declare that the research was conducted in the absence of any commercial or financial relationships that could be construed as a potential conflict of interest.

## Footnotes

^1^As surveyed by Nickels (2002), lexical retrieval models differ with respect to whether or not they assume a semantic lexicon that is separate from a semantic-conceptual system [as in models (e) and (f) in Nickels, 2002, p. 6]. Such separate semantic lexicon has been suggested by Butterworth (1989), and a similar idea can also be seen in Levelt (1989), where a distinction was suggested between a pre-verbal conceptual stage and a “mental lexicon”. We adopt such distinction on the basis of patients who show good conceptual abilities, as indicated by good comprehension of non-verbal concepts from pictures and gestures (for example, in picture association and odd one out picture tasks), who are impaired in the comprehension and production of the parallel spoken and written words (for example in word association and odd one out word tasks). Such patients were reported for example by Friedmann and Biran (2003) and Biran and Friedmann (2012).

^2^The entry in the semantic lexicon also activates the relevant entry in a syntactic lexicon (Biran and Friedmann, 2012), which we will not discuss in the current study.

^3^Another patient that was reported as showing “common mechanisms in dysnomia and post-semantic surface dyslexia” is RF, reported by Margolin et al. (1985). However, RF also was not a clear case of phonological output lexicon anomia and surface dyslexia: her error pattern in naming included only definitions but no phonological errors, so her naming deficit may have not been in the phonological output lexicon, and only 38% of her reading errors were surface errors, and she demonstrated no significant difference in reading regular and irregular words.

^4^The t-test suggested by Crawford and Howell (1998, see also Crawford and Garthwaite, 2002, 2012) allows neuropsychological studies to test the difference between a single case and a control group, and determine whether the performance of a person is significantly poorer than that of a sample of matched healthy control participants. The Crawford and Howell t-test takes care of the risk for inflated Type I errors (leading researchers to incorrectly conclude that a patient shows abnormal performance), by treating the mean and SD of the control group as statistics, namely, as belonging to a control sample rather than as known population means and SD.

^5^Good performance in the homophone-potentiophone written comprehension test (described in Section Reading Tasks without Oral Output below) is also indicative of spared lexical semantic and conceptual abilities.

^6^If the reader finds herself/himself wondering why we have not compared the reading of irregular and regular words, this is the reason: this is a luxury that only languages that do have regular words can afford.

^7^Since, the completion of this paper, we tested two additional aphasic patients with phonological lexicon anomia, a man and a woman. Both showed the same pattern as the other 16: very impaired naming with mainly phonological paraphasias and long hesitations, alongside good semantic abilities and good non-word and morphologically complex word repetition. Both showed poor reading aloud of irregular words and potentiophones, with unimpaired lexical decision of pseudohomophones and homophone comprehension.

^8^The good performance of all the participants in the lexical decision and homophone comprehension tasks also bears on the reading of the four participants who acquired Hebrew reading in their teens. It indicates that the homophones and irregular words were represented correctly in their lexicons and their surface errors in reading aloud did not result from Hebrew being their second language.

^9^We assume that such a direct route exists on the basis of reports of patients who had intact orthographic and phonological lexica and intact semantic route (good comprehension of written words including homophones, and good naming) with surface errors in reading aloud (Friedmann and Lukov, 2008; Khentov-Kraus and Friedmann, 2011), and of patients who show the opposite dissociation, with impaired semantics and good reading aloud of irregular words (Wilson and Martínez-Cuitiño, 2012).
