# Supplementary material for: A Principled Relation between Reading and Naming in Acquired and Developmental Anomia: Surface Dyslexia Following Impairment in the Phonological Output Lexicon
Source: Front Psychol. 2016 Mar 30;7:340. doi: 10.3389/fpsyg.2016.00340 (PMC4811952; doi:10.3389/fpsyg.2016.00340)
Supplement: Supplementary file 1 [file DataSheet1.docx]

**Appendix A**

Seven of the participants made additional reading errors in reading beyond their surface dyslexia errors, shown in Tables A1 and A2. Of these participants, the three men with acquired anomia, DAN, LER, and ARI, had phonological output buffer deficit (as indicated by their nonword repetition, length effect, and performance in span tasks, see Tables 2 and 3). This deficit accounts for their additional errors in reading aloud.

The four participants with developmental anomia, ARO, NIV, SHL, and SAN, made many letter transpositions within and between words, which resulted from a deficit in the orthographic-visual analyzer, in the functions of letter position encoding and letter-to-word binding. ^[[1]](#footnote-2)^ ARO and NIV did not have a phonological buffer deficit, as indicated by their good nonword repetition; SHL and SAN had both an orthographic-visual analyzer deficit and a phonological buffer deficit, as indicated by their poor nonword repetition and limited STM spans. We ran additional reading tests for these participants of reading aloud of 120 migratable words, i.e., words in which middle letter migration creates another existing word, of 60 migratable word pairs, in which a letter migration between the words create other words, and lexical decision task with nonwords created by migration of middle letters. The results of these tests, detailed in Table A3, indicate that all four of them made significantly more migrations within words than children their age, which supported their classifications as having letter position dyslexia (Friedmann & Gvion, 2001; Friedmann & Rahamim, 2007; Kohnen, Nickels, Castles, Friedmann, & Mcarthur, 2012). They accepted migratable nonwords as words, supporting the locus of their letter migrations in the orthographic-visual input, rather than the phonological output stages. All four also made significantly more between-word migrations compared to children their age, indicating that they also had attentional dyslexia (Friedmann, Kerbel, & Shvimer, 2010).

This picture also corresponds to their performance in the reading of 40 nonwords, created by migration or substitution of a letter in existing words: as summarized in Table A4, YOS, NAV, DOR, LEO, TAF, AFI, and MAD read the nonwords with 5 errors or less, and performed within the normal range for their ages in this task (or very close to the norm – the threshold for adults in this task is 4 errors), whereas the participants with phonological output buffer deficit or an orthographic-visual analyzer deficit on top of the phonological output lexicon deficit (LER, ARI, SAN, NIV, ARO, and SHL) made significantly more errors on the nonword reading task than age-matched controls.

Table A1. Error types in the TILTAN screening test of single word reading: Number of errors of each type out of 136 words each participant read^a^

| other | Consonant letter additions, omissions, substitutions | Vowel letter additions, omissions substitutions | Between word vowel letter migration | Between word consonant letter migration | Transposition of vowel letters | Transposition of consonant letters | Surface-dyslexia errors | | Participant |
| --- | --- | --- | --- | --- | --- | --- | --- | --- | --- |
| **Acquired anomia** | | | | | | | | | |
|  | 3* | 5* | 2* | 1 |  | 1* | 16* | | **DAN** |
|  | 1* |  | 1* |  |  |  | 8* | | **YOS** |
|  |  | 2* | 1* | 1 | 1* | 2* | 6* | | **ZAB** |
|  | 3* | 1* |  | 1 |  |  | 4* | | **BAR** |
| 3 | 33* | 3* | 1* | 7* | 3* | 2* | 12* | | **LER** |
| 3 | 7* |  |  | 1 | 1* |  | 25* | | **ARI** |
|  |  |  |  |  |  |  | 8* | | **NAV** |
| 1 |  | 2* |  | 1 |  | 2* | 12* | | **DOR** |
| **Developmental anomia** | | | | | | | | | |
|  |  | 1 |  |  |  | 1 | | 7* | **LEO** |
|  | 1 | 2 |  |  | 1* | 4* | | 27* | **TAF** |
|  |  |  |  |  |  | 2* | | 2 | **AFI** |
| 4* |  | 4* | 1* | 3* | 4* | 5* | | 64* | **ARO** |
|  |  |  |  |  | 2* |  | | 17* | **MAD** |
| 2 | 3 | 5* |  | 3* | 5* | 5* | | 9* | **SAN** |
|  | 1 | 1 | 1* | 2* | 2* | 5* | | 12* | **NIV** |
|  |  | 2 | 2* | 2* | 2* | 6* | | 12* | **SHL** |

* Significantly more errors than age-matched control group (*p* < .05)

^a^Dan read only 70 words; Yos read 130

NAV made no errors beside her surface errors

Table A2. Error types in the **potentiophone** reading test: number of errors of each type out of 78 words each participant read

| Semantic substitutions | Vowel letter additions, omissions substitutions | Between word vowel letter migration | Between word consonant letter migration | Consonant letter additions, omissions, substitutions | Transposition of vowel letters | Transposition of consonant letters | Surface-dyslexia errors | participant |
| --- | --- | --- | --- | --- | --- | --- | --- | --- |
| **Acquired anomia** | | | | | | | | |
|  |  |  |  | 1 |  |  | 32* | **DAN** |
|  | 1 |  |  | 1 |  |  | 12* | **YOS** |
|  | 2* |  |  | 2 | 1* |  | 17* | **ZAB** |
|  |  |  |  |  |  |  | 14* | **BAR** |
|  |  |  | 2* | 9* |  |  | 25* | **LER** |
|  |  |  |  |  |  |  | 31* | **ARI** |
|  |  |  |  |  |  |  | 10* | **NAV** |
|  |  |  |  |  |  |  | 15* | **DOR** |
| **Developmental anomia** | | | | | | | | |
|  |  |  | 1 |  |  |  | 17* | **LEO** |
|  | 1 |  |  |  |  |  | 37* | **TAF** |
|  |  |  |  |  |  |  | 16* | **AFI** |
|  | 1 |  |  |  |  |  | 36* | **MAD** |

* Significantly more errors than age-matched control group (*p* < .05)

AFI made no errors beside her surface errors

ARO, SAN, NIV, and SHL did not read this word list.

Table A3. Performance of the participants with suspected orthographic-visual analyzer deficit in letter position dyslexia and attentional dyslexia tasks

| **Lexical decision:  migratable nonwords**  Accepting a migratable nonword as an existing word | **Reading of 60 migratable word pairs**  between-word migrations | **Reading of 120 migratable words**: within-word migrations | **Participant** |
| --- | --- | --- | --- |
| 6/26 | 17/60 | 16/120 | **SAN** |
| 3/26 | 10/60 | 11/120 | **NIV** |
| 11/26 | 19/60 | 13/120 | **SHL** |
| - | 11/30 nonword pairs  4/17 word pairs | 15/60 | **ARO** |

Table A4. Number of correct responses in reading 40 nonwords

| **Nonword reading** | **Participant** |
| --- | --- |
| **Acquired anomia** | |
| 35/40 | **DAN** |
| 35/40 | **YOS** |
| 32/40 | **ZAB** |
| 38/40 | **BAR** |
| 22/40 | **LER** |
| 29/40 | **ARI** |
| 36/40 | **NAV** |
| 35/40 | **DOR** |
| **Developmental anomia** | |
| 39/40 | **LEO** |
| 35/40 | **TAF** |
| 38/40 | **AFI** |
| 32/40 | **ARO** |
| 38/40 | **MAD** |
| 14/40 | **SAN** |
| 32/40 | **NIV** |
| 25/40 | **SHL** |

1. The perfect performance of some of them in the reading input tasks— lexical decision of pseudo-homophones and comprehension of homophones and potentiophone — does not rule out letter position dyslexia or attentional dyslexia, because these tests were not sensitive to these dyslexias: they included only a single migratable word, and presented words in isolation. [↑](#footnote-ref-2)
